# Performance of Publicly Available Large Language Models on Internal Medicine Board-style Questions

**DOI:** 10.1371/journal.pdig.0000604

**Published:** 2024-09-17

**Authors:** Constantine Tarabanis, Sohail Zahid, Marios Mamalis, Kevin Zhang, Evangelos Kalampokis, Lior Jankelson

**Affiliations:** 1 Leon H. Charney Division of Cardiology, NYU Langone Health, New York University School of Medicine, New York, New York, United States of America; 2 Information Systems Laboratory, University of Macedonia, Thessaloniki, Greece; 3 Department of Internal Medicine, NYU Langone Health, New York University School of Medicine, New York, New York, United States of America; Intel Corporation, UNITED STATES OF AMERICA

## Abstract

Ongoing research attempts to benchmark large language models (LLM) against physicians’ fund of knowledge by assessing LLM performance on medical examinations. No prior study has assessed LLM performance on internal medicine (IM) board examination questions. Limited data exists on how knowledge supplied to the models, derived from medical texts improves LLM performance. The performance of GPT-3.5, GPT-4.0, LaMDA and Llama 2, with and without additional model input augmentation, was assessed on 240 randomly selected IM board-style questions. Questions were sourced from the Medical Knowledge Self-Assessment Program released by the American College of Physicians with each question serving as part of the LLM prompt. When available, LLMs were accessed both through their application programming interface (API) and their corresponding chatbot. Mode inputs were augmented with Harrison’s Principles of Internal Medicine using the method of Retrieval Augmented Generation. LLM-generated explanations to 25 correctly answered questions were presented in a blinded fashion alongside the MKSAP explanation to an IM board-certified physician tasked with selecting the human generated response. GPT-4.0, accessed either through Bing Chat or its API, scored 77.5–80.7% outperforming GPT-3.5, human respondents, LaMDA and Llama 2 in that order. GPT-4.0 outperformed human MKSAP users on every tested IM subject with its highest and lowest percentile scores in Infectious Disease (80th) and Rheumatology (99.7th), respectively. There is a 3.2–5.3% decrease in performance of both GPT-3.5 and GPT-4.0 when accessing the LLM through its API instead of its online chatbot. There is 4.5–7.5% increase in performance of both GPT-3.5 and GPT-4.0 accessed through their APIs after additional input augmentation. The blinded reviewer correctly identified the human generated MKSAP response in 72% of the 25-question sample set. GPT-4.0 performed best on IM board-style questions outperforming human respondents. Augmenting with domain-specific information improved performance rendering Retrieval Augmented Generation a possible technique for improving accuracy in medical examination LLM responses.

## Author summary

Following the recent popularization of large language models (LLMs), medical research is attempting to benchmark LLMs’ medical competency against that of practicing physicians. No published studies have investigated LLM performance on the Internal Medicine (IM) Board Examination required for physicians to certify their IM specialty. We assessed the performance of GPT-3.5, GPT-4.0, LaMDA and Llama 2, with and without additional model input augmentation, on 240 IM board-style questions sourced from the Medical Knowledge Self-Assessment Program, a preparatory question bank. GPT-4.0 scored 77.5–80.7% outperforming GPT-3.5, human respondents, LaMDA and Llama 2 in that order. GPT-4.0 outperformed human respondents on every tested IM subject with its highest and lowest percentile scores in Infectious Disease (80th) and Rheumatology (99.7th), respectively. There was an increase in test scores of both GPT-3.5 and GPT-4.0 after model input augmentation using Harrison’s Principles of Internal Medicine, a standard medical textbook. The ability to correctly answer an array of multidisciplinary questions while providing supporting explanations speaks to LLMs’ potential as a study aid and medical assistant. The ability to improve LLM test performance by model input augmentation using a standard medical textbook provides a technical approach to improving their factual accuracy.

## Introduction

Several recently released Large Language Models (LLMs) include GPT-3.5 and GPT-4.0 (OpenAI), LaMDA (Google) and Llama 2 (Meta), considered state-of-the-art due to their performance on common evaluation benchmarks (**Table 1**). The public chatbots ChatGPT, Bing Chat and Google Bard use GPT-3.5, GPT-4.0 and LaMDA, respectively [1]. There has been a surge in the proposed medical applications of LLMs, including medical note taking and consultations [2]. To that end, attempts have been made to benchmark LLM performance against practicing physicians’ fund of knowledge. Research has evaluated their performance on the United States Medical Licensing Examination [3,4] as well as the ophthalmology [1], neonatal [5] and dermatology [6] board exams. Depending on the exam in question, LLMs performed below or above passing score rates and human respondents, raising the question of LLM performance on other subject matters. Additionally, the question of improving LLM performance with additional targeted augmentations also remains since these previous studies did not enhance the LLM systems with domain specific information. In this study, we investigate the four aforementioned LLMs’ performance with and without additional enhancements, on internal medicine (IM) board-style questions from the Medical Knowledge Self-Assessment Program (MKSAP) released by the American College of Physicians [7].

**Table 1:** Large Language Model characteristics. A summary of model performance on common evaluation benchmarks along with their basic architecture and training setup, where available.

	GPT-4 [14]	GPT-3.5 [14]	LaMDA [15]	Llama2-7b [16]
MMLU	86.4% (5-shot)	70% (5-shot)	-	45.3% (5 shot)
HellaSwag	95.3% (10-shot)	85.5% (10-shot)	-	77.2% (0-shot)
WinoGrande	87.5% (5-shot)	81.6% (5-shot)	-	69.2% (0-shot)
HumanEval	67% (0-shot)	48.1% (0-shot)	-	12.8% (pass@1)
GSM-8K	92% (5-shot, CoT)	57.1% (5-shot)	-	14.6% (8-shot)
Parameters			137B	7B
Training data (tokens)			2.81T	2T

## Results

**Fig 1:**
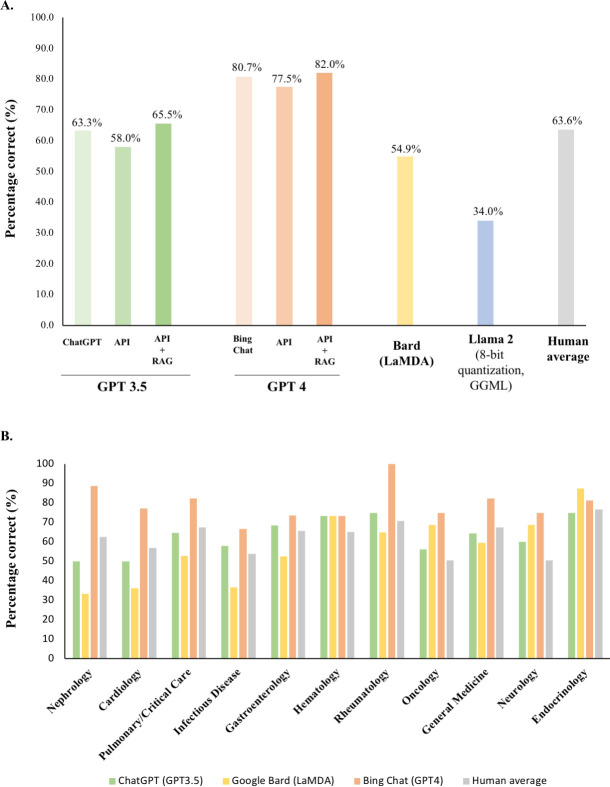
**Model performance (A)** Performance of four large language models (LLMs) evaluated by the percentage of correct answers obtained in 240 internal medicine board-style questions. Llama 2 was queried in the form of a quantized mode (8-bit quantization, GGML). **(B)** Performance evaluated by the percentage of correct answers obtained in each major subject area.

Each LLM’s performance on 240 IM board-style questions is presented in **Fig 1A**. GPT-4.0, accessed either through Bing Chat or its application programming interface (API), scored 77.5–80.7% well above the reported 64% American Board of Internal Medicine (ABIM) exam passing score, outperforming GPT-3.5, LaMDA and Llama 2 in that order. Human MKSAP users averaged 63.6%, on par with GPT-3.5 accessed through ChatGPT or its API with additional input augmentation. There is a 3.2–5.3% decrease in performance of both GPT-3.5 and GPT-4.0 when accessing the LLM through its API instead of its chatbot. There is 4.5–7.5% increase in performance of both GPT-3.5 and GPT-4.0 accessed through their APIs after additional input augmentation in the form of Harrison’s Principles of Internal Medicine [8] using the method of Retrieval Augmented Generation.

**Fig 1B** depicts the performance in each IM subject of the top three highest scoring LLMs accessed through their corresponding chatbots. Similar to overall performance, GPT-4.0 outperformed human MKSAP users on every tested IM subject. The percentile performance of GPT-4.0 for each IM subject according to a nomogram of human MKSAP users is listed in **Table 2**. The highest and lowest percentile scores were in Infectious Disease (80th) and Rheumatology (99.7th), respectively. Additionally, GPT-4.0 outperformed both GPT-3.5 and LaMDA on all IM subjects with the exception of endocrinology and hematology. In nearly every IM subject, GPT-3.5 (ChatGPT) outperformed LaMDA (Bard), with the exception of Oncology and Neurology.

**Table 2:** Benchmark against human test takers. Performance of Bing Chat (GPT-4.0) on internal medicine board-style questions in each major subject area expressed as a percentile among human test takers.

Specialty	Percent Correct	Percentile
Nephrology	88.9	99.5
Cardiology	77.3	92.2
Pulmonary/Critical Care	82.4	94.3
Infectious Disease	66.7	80
Gastroenterology	73.7	97.2
Hematology	73.3	88.2
Rheumatology	100	>99.7
Oncology	75	93.2
General Medicine	82.3	94.3
Neurology	75	96.9
Endocrinology	81.3	94.7

An IM board-certified, blinded reviewer correctly identified the human generated MKSAP response over the GPT-3.5 (ChatGPT) and GPT-4.0 (Bing Chat) responses in 72% of a 25-question sample set. In all but one of the incorrectly identified cases, the reviewer picked ChatGPT as the human response. The reviewer cited a writing style geared towards laypeople rather than IM residents and a comparatively greater number of references to IM subspecialty society guidelines, as the two primary ways of identifying the LLM-generated response. Factual accuracy and clinical reasoning reportedly did not inform the reviewer’s decision.

## Discussion

The LLMs and in particular GPT-4.0, exhibited high overall and subject-based performance, even without additional input augmentation, while their explanations contained valuable information from a medical education standpoint. Accessed through its API or corresponding chatbot, GPT-4.0 consistently outperformed human respondents and could be reasonably expected to pass the ABIM exam. The present study’s limitations include the inability to test ABIM questions directly and the use of solely text-based questions.

The drop in performance of both GPT-3.5 and GPT-4.0 when accessing the LLM through its API instead of its chatbot is a key finding of this study. Differences in the underlying LLM system and/or the degree of model training between the API and chatbot versions could explain this observation but are difficult to ascertain given the “black box” nature of these models.[9] Regardless, the drop in LLM performance via the API was recovered through model input enhancements in the form of Harrison’s Principles of Internal Medicine [8] for both GPT-3.5 and GPT-4.0. This equivalent performance is key, since an application built upon the LLM’s API would not necessarily require internet access limiting ongoing concerns for loss of patient privacy or other sensitive information. Additionally, the 4.5–7.5% score increase following input augmentation with a relatively limited amount of domain specific information raises the possibility of greater improvements with additional augmentation. High yield, structured, domain specific information to assist with this task can be derived from publicly accessible IM subspecialty society websites (e.g., American College of Cardiology), which include updated practice guidelines and clinical trial summaries among other content. The subject-based performance (**Fig 1B**) analysis can further inform targeted LLM input enhancement. Model input augmentation can be focused on the worst performing subjects to ensure comparatively large improvements in overall examination performance.

Each question answered incorrectly by an LLM can be considered a “hallucination.”[2] Such “hallucinations” are stated in an assertive manner raising concerns of convincing human users of an erroneous answer. On the one hand, the LLMs failed a rudimentary “Turing”-like test as the blinded reviewer identified more than 50% of the human generated responses. On the other hand, the 28% failure rate provides further support regarding the similarity between LLM- and human-generated responses. To address this, ascribing a variable degree of confidence to each LLM response is not possible with current LLM designs, as the models were trained to emulate textual patterns rather than weigh each generated response according to their degree of factual accuracy [2,9]. Bing Chat adds the caveat that the response is AI-generated and further consultation with experts is needed. Additionally, its provision of references allows human users to fact check its responses. The aforementioned illustration of performance improvement with model input augmentation using domain specific information (**Fig 1A**) could address this limitation by improving factual accuracy.

Despite its limitations, this study provides insights into the future application of LLMs to medical practice. The ability to answer an array of multidisciplinary IM questions while providing supporting explanations speaks to LLMs’ potential as a study aid and medical assistant during hospital rounds. Additionally, the ability to reverse the drop in LLM performance when accessing its API through input augmentation with domain specific information will be key when developing LLM-based medical applications that seek to ensure patient privacy. Yet, we should be wary of possible improper uses by limiting access to such artificial intelligence tools during open-book ABIM assessments among physicians maintaining their ABIM board certification.

## Materials and methods

### Question selection

MKSAP questions are similar in complexity to the multiple-choice questions from the ABIM standardized exam, spanning a broad scope of knowledge from diagnosis to medical management. MKSAP is commonly used by IM residents to prepare for the ABIM exam. The ABIM has no publicly available testing material and declined a formal request to release sample questions. In addition to MKSAP, the ACP administers the in-training exam (ITE) to IM residents, a yearly multiple-choice standardized exam used to track residents’ educational growth. Performance on the ITE during the third year of IM residency is an independent predictor for passing the ABIM examination [10]. The MKSAP questions included in this study were released after January 2022, outside the scope of training data in GPT-3.5 and GPT-4.0, which was confirmed by a Google index search prior to January 1, 2022. All questions containing clinical images were excluded, given the LLMs’ inability to process visual information at the time of study. A random selection of 240 questions reflected the number of questions in an ABIM examination and comprised of all major subject areas (representing at least 4% of the ABIM exam [11]). Google and Bing web searches of each of the selected question prompts did not reveal any matching results.

### Performance evaluation

All LLM queries took place between July 1^st^ and September 1^st^, 2023. Three LLMs (GPT-3.5, GPT-4.0 and LaMDA) were accessed through their aforementioned publicly available chatbots. Each question was asked in a new chat with a cleared history. GPT-3.5 and GPT-4.0 were also accessed through the OpenAI API, specifically models gpt-3.5-turbo-16k-0613 and gpt-4-0613, respectively. A quantized version of LlaMA-2-chat-7b (8-bit, GGML quantization) was hosted and accessed locally. The OpenAI API models were called at zero temperature to ensure maximal model output consistency while no conversational memory was retained between questions. These models were also additionally enhanced by providing context in the form of Harrison’s Principles of Internal Medicine [8] through the method of Retrieval Augmented Generation (RAG) [12]. Using this method, the model input is expanded to include parts of the provided document that are semantically similar to the exam question posed [13]. More specifically, the first 170 pages of Harrison’s Principles of Internal Medicine were discarded, as the information contained within was not relevant to the subject (editor information, table of contents, etc.). The filtered document was subsequently split recursively on certain characters (double and single newline character and space) in chunks of 750 characters with 150-character overlap. For each derived chunk, its embeddings were calculated using the text-embedding-ada-002 embedding model (OpenAI) and stored in a Chroma vector database. For each question, 90 of the most similar documents to the question were retrieved from the database to be passed as context. The document-question similarity metric used was cosine similarity. Both question and context were passed to the model wrapped in the following prompt:

“`Use the following pieces of context to answer the question at the end. If you don’t know the answer, just say that you don’t know, don’t try to make up an answer.

{context}

Question: {question}

Helpful Answer: “`

The RAG prompt used was the same as the one used for the simple API calls, with the addition of context-related instructions. Each question served as part of the LLM prompt, while both an incorrect answer and a declination to answer were considered incorrect. Separately, explanations to 25 correctly answered questions generated by ChatGPT and Bing Chat were randomly selected. The explanations were presented in a blinded fashion alongside the human generated MKSAP explanation to an IM board-certified physician, who had not previously used MKSAP. The blinded reviewer was tasked with selecting the human generated explanation.

## Conclusions

Overall, GPT-4.0 performed best among the studied LLMs on IM board-style questions, outperforming human respondents and demonstrating promise for future medical applications. Augmentation with a limited amount of domain-specific information improved LLM performance rendering Retrieval Augmented Generation a viable technique for improving factual accuracy in medical examination LLM responses.

## References

[1] Cai LZ, Shaheen A, Jin A, Fukui R, Yi JS, Yannuzzi N, et al. Performance of Generative Large Language Models on Ophthalmology Board–Style Questions. Am J Ophthalmol. 2023;254: 141–149. doi: 10.1016/j.ajo.2023.05.024 37339728

[2] Lee P, Bubeck S, Petro J. Benefits, Limits, and Risks of GPT-4 as an AI Chatbot for Medicine. Drazen JM, Kohane IS, Leong T-Y, editors. N Engl J Med. 2023;388: 1233–1239. doi: 10.1056/NEJMsr2214184 36988602

[3] Gilson A, Safranek CW, Huang T, Socrates V, Chi L, Taylor RA, et al. How Does ChatGPT Perform on the United States Medical Licensing Examination? The Implications of Large Language Models for Medical Education and Knowledge Assessment. JMIR Med Educ. 2023;9: e45312. doi: 10.2196/45312 36753318 PMC9947764

[4] Kung TH, Cheatham M, Medenilla A, Sillos C, De Leon L, Elepaño C, et al. Performance of ChatGPT on USMLE: Potential for AI-assisted medical education using large language models. Dagan A, editor. PLOS Digit Heal. 2023;2: e0000198. doi: 10.1371/journal.pdig.0000198 36812645 PMC9931230

[5] Beam K, Sharma P, Kumar B, Wang C, Brodsky D, Martin CR, et al. Performance of a Large Language Model on Practice Questions for the Neonatal Board Examination. JAMA Pediatr. 2023;177: 977. doi: 10.1001/jamapediatrics.2023.2373 37459084 PMC10352922

[6] Mirza FN, Lim RK, Yumeen S, Wahood S, Zaidat B, Shah A, et al. Performance of Three Large Language Models on Dermatology Board Examinations. J Invest Dermatol. 2023. doi: 10.1016/j.jid.2023.06.208 37541614

[7] ACP MKSAP 19. Available: https://www.acponline.org/featured-products/mksap-19.

[8] Loscalzo J, Fauci A, Kasper D, Hauser S, Longo D, Jameson LJ. Harrison’s Principles of Internal Medicine. McGraw Hill / Medical; 2022.

[9] Liesenfeld A, Lopez A, Dingemanse M. Opening up ChatGPT: Tracking openness, transparency, and accountability in instruction-tuned text generators. Proceedings of the 5th International Conference on Conversational User Interfaces. New York, NY, USA: ACM; 2023. pp. 1–6. doi: 10.1145/3571884.3604316

[10] Rayamajhi S, Dhakal P, Wang L, Rai MP, Shrotriya S. Do USMLE steps, and ITE score predict the American Board of Internal Medicine Certifying Exam results? BMC Med Educ. 2020;20: 79. doi: 10.1186/s12909-020-1974-3 32183789 PMC7079442

[11] Blueprint for the Internal Medicine MOC Exam. Available: https://www.abim.org/maintenance-of-certification/assessment-information/internal-medicine/exam-content.

[12] Lewis P, Perez E, Piktus A, Petroni F, Karpukhin V, Goyal N, et al. Retrieval-Augmented Generation for Knowledge-Intensive NLP Tasks. Adv Neural Inf Process Syst. 2020; 9459–9474. Available: http://arxiv.org/abs/2005.11401.

[13] Cai D, Wang Y, Liu L, Shi S. Recent Advances in Retrieval-Augmented Text Generation. Proceedings of the 45th International ACM SIGIR Conference on Research and Development in Information Retrieval. New York, NY, USA: ACM; 2022. pp. 3417–3419. doi: 10.1145/3477495.3532682

[14] OpenAI, Achiam J, Adler S, Agarwal S, Ahmad L, Akkaya I, et al. GPT-4 Technical Report. 2023. Available: http://arxiv.org/abs/2303.08774.

[15] Thoppilan R, De Freitas D, Hall J, Shazeer N, Kulshreshtha A, Cheng H-T, et al. LaMDA: Language Models for Dialog Applications. 2022. Available: http://arxiv.org/abs/2201.08239.

[16] Touvron H, Martin L, Stone K, Albert P, Almahairi A, Babaei Y, et al. Llama 2: Open Foundation and Fine-Tuned Chat Models. 2023. Available: http://arxiv.org/abs/2307.09288.

